# Hypermucoviscous Klebsiella pneumoniae: A Hypervirulent Strain Masquerading as Metastasis

**DOI:** 10.7759/cureus.39561

**Published:** 2023-05-27

**Authors:** Mulham Ombada, Talha Perwez, Marco Campitelli, Nabil Zeineddine

**Affiliations:** 1 Internal Medicine, State University of New York Upstate Medical University, Syracuse, USA; 2 Infectious Diseases, State University of New York Upstate Medical University, Syracuse, USA; 3 Pulmonary/Critical Care, Samaritan Medical Center, Watertown, USA

**Keywords:** severe sepsis, splenic abscess, perigastric abscess, hepatic abscess, hypervirulent klebsiella pneumoniae

## Abstract

Hypermucoviscous Klebsiella pneumoniae (HvKP) is a hypervirulent strain of Klebsiella that causes metastatic spread and life-threatening infection. While it is more common among people of Asian descent, it has been increasingly reported globally among people of other ethnicities as well. We report a case of pan-susceptible HvKP infection in a male patient of Asian descent who has been living in the US for 20 years. It caused a liver abscess, perigastric abscess, perisplenic abscess, multifocal pneumonia, septic emboli, and tricuspid valve infective endocarditis. He was treated with ceftriaxone, but his septic shock was refractory, ultimately leading to death. This case highlights the severity of infection caused by this strain, and its ability to present with radiographic signs suggestive of malignancy with metastasis. This case also suggests that this strain can become pathogenic after a very prolonged period of gastrointestinal colonization.

## Introduction

Metastatic spread is an unusual feature for enteric gram-negative bacilli, particularly in non-immunocompromised individuals. Hypervirulent Klebsiella pneumoniae - also known as hypermucoviscous Klebsiella pneumoniae (HvKP) - has emerged as an exception to this rule. While it was first described in the Asia-Pacific region, HvKp is now being increasingly recognized globally. This strain might be more common in patients of Asian descent due to genetic susceptibility rather than geo-specific acquisition, but this has not been confirmed. Along with its hypervirulence, this strain can acquire extensive antimicrobial resistance. This, along with its global spread, makes it a potential cause of devastating morbidity and mortality worldwide. Going forward, this strain needs to be considered when empirically treating devastating infections, including endocarditis, meningitis, and endophthalmitis in the appropriate clinical and epidemiological setting [1-3].

## Case presentation

The patient was a male of Asian descent in his 60s, previously healthy, who presented to the emergency room via EMS for five days of fever associated with malaise, vomiting, and decreased appetite. The patient was immunocompetent but with no routine medical follow-up. His social history revealed that he consumed alcohol daily. He had moved to the US 20 years ago. Upon admission, the patient was noted to have hyperglycemia, hypotension, and hypoxic respiratory failure. His physical exam was positive for scleral icterus, abdominal tenderness, hepatomegaly, and lower extremities edema. He was admitted for septic shock.

Investigations

**Figure 1:**
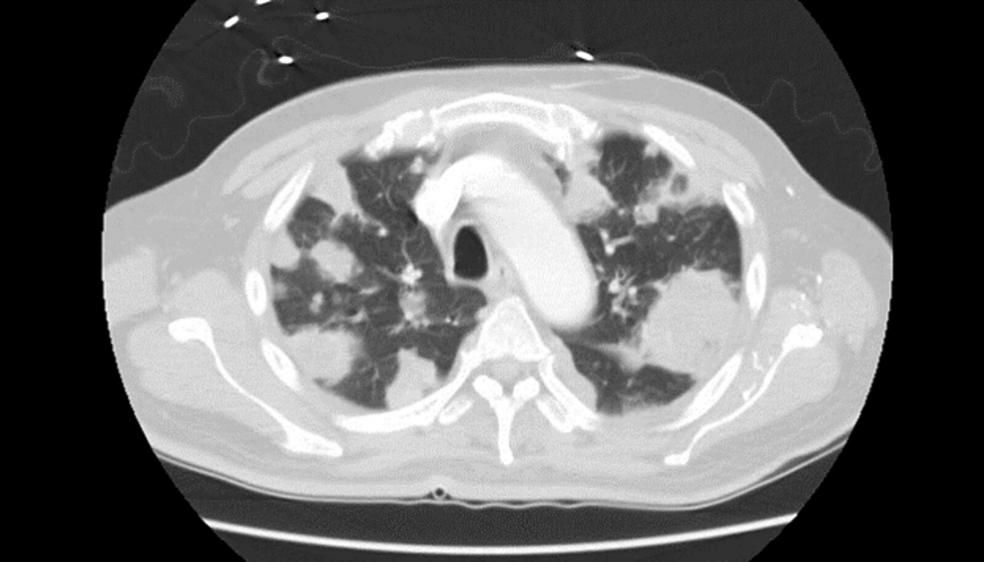
Coronal cut of CT chest showing multiple rounded masses and consolidations CT: computed tomography

Laboratory workup revealed leukocytosis with a WBC count of 17600/Ul, high anion gap acidosis with a blood glucose of 544 mg/dl, corrected sodium of 127 meq/l, elevated total bilirubin at 6.6 mg/dl, and direct component of 5.2 mg/dl. His aspartate aminotransferase (AST) and alanine aminotransferase (ALT) levels were within normal limits. EKG showed ST elevation in multiple leads with no regional wall motion abnormalities on echocardiography, suggestive of pericarditis. CT abdomen and pelvis showed a left hepatic lobe hypodense lesion and complex fluid collections in the left upper quadrant adjacent to the spleen with a mass effect on the stomach, suggestive of malignancy. CT chest showed multiple rounded masses and consolidations in both lungs with complex left-side pleural effusion, also suggestive of malignancy (Figure 1).

Differential diagnosis

Due to concerns for malignancy with multiple metastases, percutaneous sampling of the liver lesion was done, which yielded a thick white material. This procedure was followed by percutaneous drain placement in perigastric and perisplenic fluid collections, yielding pus. Cultures of all these samples, along with blood and sputum cultures, grew Klebsiella pneumoniae. Transthoracic echocardiography findings were limited but showed very severe tricuspid regurgitation, establishing the diagnosis of tricuspid valve endocarditis based on modified Duke Criteria. An ophthalmological evaluation was negative for signs of endophthalmitis or retinal emboli.

**Figure 2:**
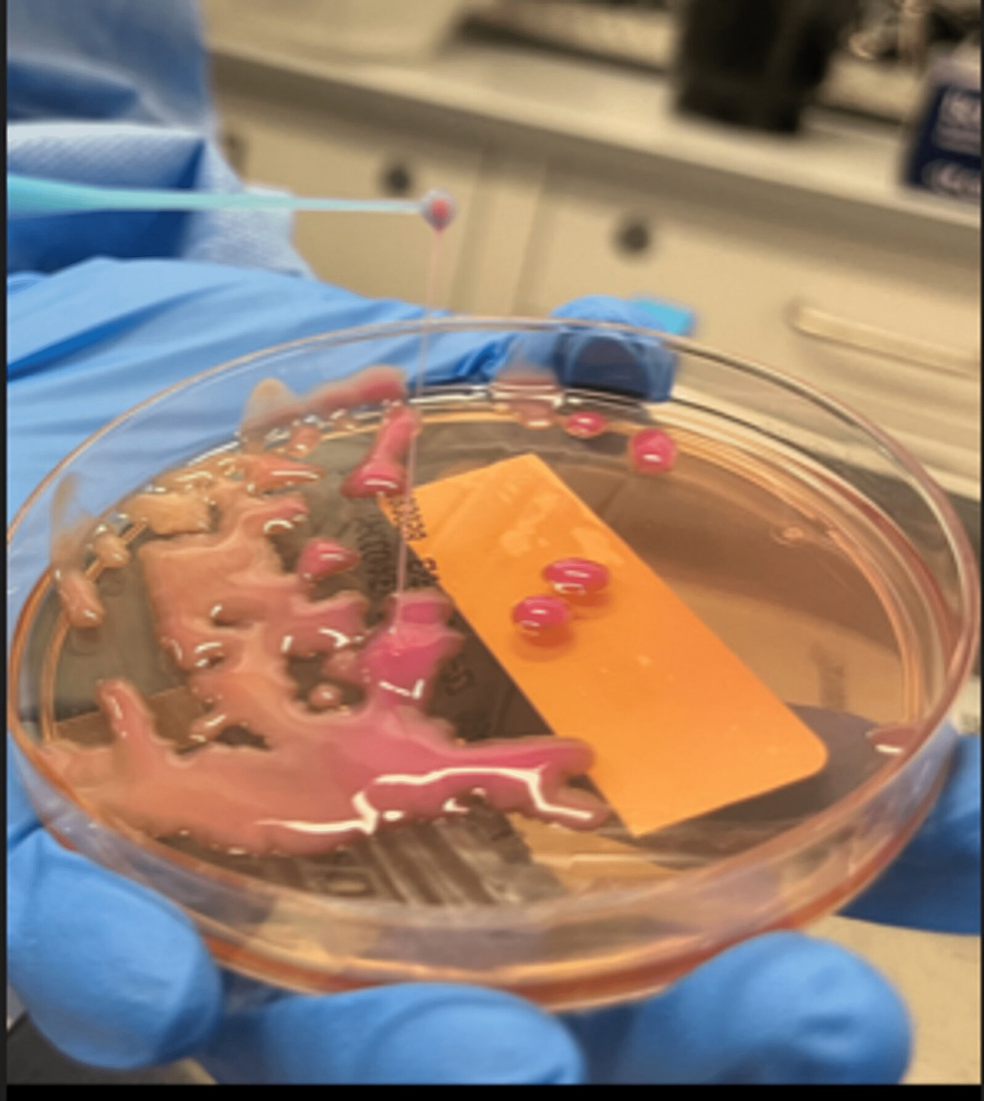
String test The string was more than 10 cm in length (positive test defined as >5 mm)

A string test was done to evaluate for hypermucoviscous Klebsiella strain and was clearly positive (Figure 2).

Treatment

The patient received resuscitation with intravenous fluids, an insulin drip for diabetic ketoacidosis (DKA), and a new diagnosis of diabetes. He was diagnosed with shock and started on norepinephrine and empiric vancomycin and piperacillin-tazobactam IV.

Klebsiella pneumoniae was pan-susceptible with a minimum inhibitory concentration (MIC) <1 to ceftriaxone (Table 1). The patient was transitioned to ceftriaxone 2 g IV q24h.

**Table 1:** Antimicrobial susceptibility of the Klebsiella pneumoniae isolate MIC: minimum inhibitory concentration

	Klebsiella pneumoniae	
	Select MIC results reported	Select Kirby-Bauer results reported
Ampicillin + sulbactam	8/4 Sensitive	
Cefazolin		Sensitive
Ceftazidime	<=1 Sensitive	
Ceftriaxone	<=1 Sensitive	
Ciprofloxacin	<=0.25 Sensitive	
Gentamicin	<=1 Sensitive	
Levofloxacin	<=0.12 Sensitive	
Piperacillin + tazobactam	<=4 Sensitive	
Tobramycin	<=1 Sensitive	
Trimethoprim + sulfamethoxazole	<=1/19 Sensitive	

Outcome and follow-up

The patient's hospital course was complicated by acute kidney injury requiring renal replacement therapy, limb ischemia, and persistent shock, ultimately leading to his demise.

## Discussion

Metastatic spread is an unusual feature for enteric gram-negative bacilli, particularly in non-immunocompromised patients. HvKP has emerged as an exception to this rule. After first being described in the Asia-Pacific region, HvKP is now increasingly recognized globally [1]. Its clinical features are defined by its ability to cause life-threatening, community-acquired infections in immunocompetent, and sometimes, young hosts, including liver abscess, pneumonia, meningitis, endophthalmitis, and endocarditis, many of which were confirmed in our patient. The characteristics that enhance its virulence and distinguish it from other variants of Klebsiella include its thick capsule, its hypermucoviscous phenotype and immune evasion, and its increased ability to acquire iron [2].

Despite the fact that most patients affected by this hypervirulent strain are Asians, HvKP is now encountered in Western countries as well. The racial distribution of this disease might represent a genetic predisposition or simply a geo-specific strain acquisition. Our patient had not left the US for 20 years. It is difficult to distinguish a more recent acquisition of the strain from chronic enteric colonization. Serotyping and molecular testing were not done in our case, but a string test (as shown in Figure 2) was clearly positive, with a thread of more than 10 cm [3]. The susceptibility of HvKP strain to antimicrobials is variable, ranging from pan-susceptible, as in this case, to multidrug-resistant. Increased antibiotic use and the emergence of advanced multidrug resistance in strains like the ones we describe will undoubtedly lead to devastating rates of morbidity and mortality [4].

Early recognition of HvKP metastatic syndrome is also a key factor and should lead to timely empiric coverage, in the appropriate clinical and epidemiological setting, including coverage for multidrug-resistant strains. In our patient, initial imaging was suggestive of metastatic malignancy, though this did not delay appropriate antimicrobial use [5]. Recognition of this strain, its metastatic spread, and its global dissemination might cause a major epidemiological shift that needs to be accounted for when diagnosing and empirically treating infections such as endophthalmitis, meningitis, and infective endocarditis where enteric gram-negative bacilli are usually not a common culprit [6].

## Conclusions

HvKP causes metastatic infections and is associated with extremely high mortality rates. It is more common in patients of Asian descent, but its more recent global spread sounds the alarm for a potentially devastating impact worldwide. Our patient had a severe infection suggesting either local spread or that this strain can colonize the gastrointestinal tract for years before causing its devastating effects.

## References

[1] Shon AS, Bajwa RP, Russo TA (2013). Hypervirulent (hypermucoviscous) Klebsiella pneumoniae: a new and dangerous breed. Virulence.

[2] Walker KA, Miller VL (2020). The intersection of capsule gene expression, hypermucoviscosity and hypervirulence in Klebsiella pneumoniae. Curr Opin Microbiol.

[3] Namikawa H, Oinuma KI, Yamada K, Kaneko Y, Kakeya H, Shuto T (2023). Differences in severity of bacteraemia caused by hypermucoviscous and non-hypermucoviscous Klebsiella pneumoniae. Int J Antimicrob Agents.

[4] Zhan L, Wang S, Guo Y (2017). Outbreak by hypermucoviscous Klebsiella pneumoniae ST11 isolates with carbapenem resistance in a tertiary hospital in China. Front Cell Infect Microbiol.

[5] Sánchez-López J, García-Caballero A, Navarro-San Francisco C (2019). Hypermucoviscous Klebsiella pneumoniae: a challenge in community acquired infection. IDCases.

[6] Li W, Sun G, Yu Y (2014). Increasing occurrence of antimicrobial-resistant hypervirulent (hypermucoviscous) Klebsiella pneumoniae isolates in China. Clin Infect Dis.

